# Genetic Risk Score Predicts Late-Life Cognitive Impairment

**DOI:** 10.1155/2015/267062

**Published:** 2015-08-23

**Authors:** Mariegold E. Wollam, Andrea M. Weinstein, Judith A. Saxton, Lisa Morrow, Beth Snitz, Nicole R. Fowler, Barbara L. Suever Erickson, Kathryn A. Roecklein, Kirk I. Erickson

**Affiliations:** ^1^Department of Psychology, University of Pittsburgh, Sennott Square, 3rd Floor, 210 South Bouquet Street, Pittsburgh, PA 15260, USA; ^2^Center for the Neural Basis of Cognition, University of Pittsburgh, 4400 Fifth Avenue, Suite 115, Pittsburgh, PA 15213, USA; ^3^Department of Neurology, University of Pittsburgh, 811 Kaufmann Medical Building, 3471 Fifth Avenue, Pittsburgh, PA 15213, USA; ^4^Department of Psychiatry, University of Pittsburgh, Thomas Detre Hall, 3811 O'Hara Street, Pittsburgh, PA 15213, USA; ^5^Division of General Internal Medicine and Geriatrics, Indiana University, Fifth Third Faculty Building, 720 Eskenazi Avenue, Indianapolis, IN 46202, USA

## Abstract

*Introduction*. A family history of Alzheimer's disease is a significant risk factor for its onset, but the genetic risk associated with possessing multiple risk alleles is still poorly understood. *Methods*. In a sample of 95 older adults (Mean age = 75.1, 64.2% female), we constructed a genetic risk score based on the accumulation of risk alleles in *BDNF, COMT,* and *APOE*. A neuropsychological evaluation and consensus determined cognitive status (44 nonimpaired, 51 impaired). Logistic regression was performed to determine whether the genetic risk score predicted cognitive impairment above and beyond that associated with each gene. *Results*. An increased genetic risk score was associated with a nearly 4-fold increased risk of cognitive impairment (OR = 3.824, *P* = .013) when including the individual gene polymorphisms as covariates in the model. *Discussion*. A risk score combining multiple genetic influences may be more useful in predicting late-life cognitive impairment than individual polymorphisms.

## 1. Introduction

A family history of late-life cognitive impairment is a well-recognized risk factor for the development of Alzheimer's disease (AD) and its preclinical stage of Mild Cognitive Impairment [1] (MCI). Twin studies report the heritability of AD to be between 58 and 79% [2], prompting a persistent search for genetic variants that influence susceptibility for AD and cognitive decline. The primary genetic risk factor for late-onset AD (LOAD) is widely considered to be the *ε*4 allele of the apolipoprotein E (*APOE*) gene, which has been consistently identified by genome-wide association studies (GWAS) of AD [3]. In the brain, apolipoprotein E serves a vital function in neuronal activity by regulating cholesterol metabolism [4]. Approximately 40% of all Caucasian adults who develop AD possess at least one *ε*4 allele [5], and *ε*4 carriers exhibit more precipitous cognitive decline [6, 7].

In addition to* APOE*, other genetic variants have been identified as risk factors for cognitive decline because they influence biological pathways involved in memory and executive function, which exhibit the earliest deficits during the progression of AD [8]. For example, the* BDNF* gene codes for brain-derived neurotrophic factor, a protein secreted by neurons that promotes neurogenesis and synaptic plasticity and transmission in the central nervous system; BDNF is particularly critical for learning and memory processes because it facilitates long-term potentiation in hippocampal neurons [9]. AD has been associated with decreased BDNF levels in the temporal and parietal cortices [10]. BDNF also circulates and can be measured in the periphery. Decreased levels of serum BDNF have been associated with AD [11]. Age-related reductions in serum BDNF levels have also been related to smaller hippocampal volume and poorer memory function even in healthy older adults [12]. In the human* BDNF* gene, variation in the protein's function has been attributed to a single nucleotide polymorphism (SNP, rs6265,* Val66Met*) resulting in a* valine* (*Val*) to* methionine* (*Met*) substitution at codon 66. The* Met*-substituted product is associated with impaired intracellular trafficking and secretion of BDNF, poorer performance in declarative memory [13] and executive function [14], and reduced hippocampal volume and function [13–16]. Despite these convincing links with AD, weak association between this SNP and AD has been identified by GWAS [17] and candidate gene studies yield inconsistent results regarding whether the* Met* allele confers an increased risk of AD [18, 19], suggesting a need for further investigation of its role as a contributing, but not sole, risk factor.

In addition to* APOE* and* BDNF*, the catechol-o-methyltransferase (*COMT*) gene codes for an enzyme responsible for catalysis and inactivation of catecholamine neurotransmitters such as dopamine, norepinephrine, and epinephrine [20]. A common SNP of* COMT* (rs4680,* Val158Met*) involves a substitution of* Val* by* Met* at codon 158, the product of which is four times less metabolically active than the homozygous* Val* allele product [21]. Slower enzymatic activity of COMT delays inactivation of dopamine in the synaptic cleft in the prefrontal cortex, resulting in enhanced executive function for* Met* carriers relative to* Val* homozygotes [22]. Although association between the* COMT* polymorphism and AD has not been confirmed by GWAS or meta-analysis [23], studies have demonstrated that throughout adulthood the* Val* allele is associated with characteristics of cognitive decline and dementia such as poorer performance on tasks of executive functioning and working memory [24], declarative memory [25], and slower processing speed [26]. As in the case of BDNF* Val66Met*, the COMT* Val158Met* SNP is likely an under-recognized contributing genetic risk factor in the development of AD.

These and other genetic polymorphisms likely contribute relatively small independent effects to collectively predispose one to develop a complex disease such as AD. Studies have begun to investigate how multiple genetic influences can be aggregated into a single risk profile to predict the prevalence or course of a given pathology, either by summing the total number of risk alleles possessed or by obtaining a weighted sum including each risk allele multiplied by its associated effect size. Rodríguez-Rodríguez et al. [27] constructed a genetic risk score to predict progression from MCI to AD that combined genotype information across 8 non-*APOE* genetic variants (16 total alleles) identified by GWAS of AD risk, with each allele weighted by its AD risk odds ratio. Although the weighted genetic risk score was not significant, the authors found that subjects who possessed a total of six or more risk alleles progressed from MCI to AD twice as quickly as those who possessed fewer than six risk alleles. While the accumulation of risk alleles was a significant predictor for rate of progression to AD (OR = 1.89, *P* < .047, and 95% C.I. = 1.01, 3.56), each individual genetic polymorphism did not have significant predictive power by itself, with the exception of one marginally significant gene (*CD2AP*, OR = 1.69, *P* < .051). Similar risk scores have been employed to predict other pathologies such as age-related macular degeneration, multiple sclerosis, and type II diabetes [28–30]. These studies indicate that incorporating multiple SNPs pertinent to a given phenotype into a genetic risk score is more useful in predicting the prevalence or progression of a disease than considering polymorphisms individually.

In this genetic risk score study, we took a candidate gene approach by targeting genetic variants that have either been identified as having a clear link with risk for AD (i.e.,* APOE*) or that have an important role in cognitive and brain functions in late adulthood and have a relatively common minor allele frequency (i.e.,* BDNF*,* COMT*) but may have been previously undetected by GWAS due to small individual effect sizes. To test whether the combination of these three risk genotypes was collectively predictive of late-life cognitive impairment, we created a cumulative genetic risk score by summing the possession or absence of each SNP's risk allele. We predicted that a higher genetic risk score would correspond to an increased risk of cognitive impairment above and beyond that of the individual gene polymorphisms.

## 2. Methods

### 2.1. Participants

Data for this study were collected as part of a larger study examining the utility of providing cognitive testing of older adults in primary care physician (PCP) offices [31, 32]. Participants included 109 adults who were consented into the parent study. The parent study recruited participants from eleven PCP practices in the greater Pittsburgh and surrounding areas. Participants were eligible to participate if they were 65 years or older, had no medical chart diagnosis of dementia, had no acute illness, and were not residing in a nursing home. They were excluded if initial screening revealed the presence of sensory deficits that would preclude computerized and paper and pencil neuropsychological testing. Additionally, they were excluded if initial screening revealed the presence of dementia as indicated by a score of ≤18 on the Mini-Mental State Examination [33].

One participant was removed due to incorrectly recorded cognitive data. Since distributions of allelic frequencies differ between races, 4 African American participants were removed from the analysis to minimize confounding by genetic admixture. Age, gender, and years of education were self-reported. All participants signed a consent form approved by the University of Pittsburgh Institutional Review Board and were remunerated $20 for participation.

### 2.2. Cognitive Assessment and Group Classification

Each participant completed a comprehensive neuropsychological test battery assessing five primary cognitive domains: memory, executive function, spatial ability, language, and attention/psychomotor speed. Measures of memory were the Consortium to Establish a Registry for Alzheimer's Disease (CERAD) Word List Learning Test with delayed recall [34], the Wechsler Memory Scale-Revised Logical Memory I and II [35], and the modified Rey-Osterrieth figure for immediate and delayed recall [36]. Tests of executive function were the Wechsler Adult Intelligence Scale-Revised (WAIS-R) Backward Digit Span [37], the controlled oral word association test (FAS) [38], Part B of the Trail-Making Test [39], the WAIS-R Digit Symbol [37], and the Clock Drawing Test [38]. Tests of spatial ability were the modified Rey-Osterrieth Copy [36] and the modified WAIS-R Block Design [37]. Language tests consisted of the Boston Naming Test [40] and semantic fluency (animals) [38]. Tests of attention/psychomotor speed were the WAIS-R Digit Span Forward [37] and Part A of the Trail-Making Test [39]. Participants were also administered the Center for Epidemiologic Studies Depression (CES-D) Scale[41] for assessment of depression symptomatology, the Activities of Daily Living (ADL) Scale [42], and the Instrumental Activities of Daily Living (IADL) Scale [43].

A clinical adjudication panel of three expert neuropsychologists (all licensed psychologists) determined cognitive status: normal, mild cognitive impairment (MCI), or dementia. Classifications were done according to the criteria from the University of Pittsburgh Alzheimer's Disease Research Center [44, 45]. Dementia range = scores ≥ 2SD below age norms on two cognitive domains, one of which must be memory; MCI range = at least two scores 1-2SD below age norms; normal cognition = individuals not included in ranges for either dementia or MCI. The final diagnosis took into account the cognitive test scores as well as demographic, functional, behavioral, and medical information. Adjudications were conducted blind to study group status of the parent study. Final cognitive status categories consisted of cognitively nonimpaired (*n* = 44), Mild Cognitive Impairment (MCI, *n* = 47), or dementia (*n* = 4). Because of the low number of individuals with dementia, those with either Mild Cognitive Impairment or dementia were combined into a single group labeled “cognitive impairment” (*n* = 51).

### 2.3. Genotype Collection, Coding, and Risk Score Computation

Genomic DNA was collected with the Oragene-DNA Self-Collection Kit OG-500 (DNA Genotek Inc., Ontario, Canada). Extraction and purification of DNA were completed using the laboratory protocol from Oragene-DNA. DNA was diluted with TE buffer to 10 ng/*μ*L and stored at −20°C.

Participants were genotyped for rs6265 (*Val66Met*) in* BDNF*, rs4680 (*Val158Met*) in* COMT*, and rs429358/rs7412 in* APOE* (*ApoE2*,* ApoE3*, and* ApoE4*). Genotype analysis was performed by high-resolution melting (HRM) analysis. Each polymerase chain reaction (PCR) was performed on a CFX96 real-time PCR system (Bio-Rad, Hercules, CA) using 1x Precision Melt Supermix (Bio-Rad, Hercules, CA). The reaction volume was 20 *μ*L and contained 50 ng of genomic DNA. See Table 1 for primer sequence, primer concentration, amplicon length, and melt range. The running conditions for* BDNF* were 1 cycle of 95°C for 2 min and 65 cycles of 95°C for 10 s, and 56.6°C for 30 sec. The running conditions for* COMT* were 1 cycle of 95°C for 2 min and 65 cycles of 95°C for 10 s, and 60.1°C for 30 sec. The conditions for* APOE* were 1 cycle of 95°C for 2 min and 60 cycles of 95°C for 10 s, 63.8°C for 30 s, and 72°C for 30 sec. The melting range varied per SNP (see Table 1) but all began with a heteroduplex formation of 95°C for 30 seconds and 60°C for 1 min. The temperature increment for melting analysis for all three genotypes was 0.2°C per 10 sec. The melting curve was analyzed using Bio-Rad Precision Melt Analysis 1.2 software. Negative and positive controls were included on each run to ensure genotyping accuracy. Greater than 50% of samples were run in duplicate.

For the individual genotype analyses,* BDNF* genotype groups consisted of* Val* homozygotes,* Val* heterozygotes, and* Met* homozygotes*; COMT* genotype groups similarly consisted of* Val* homozygotes,* Val* heterozygotes, or* Met* homozygotes*; APOE* genotype groups included *ε*2/*ε*3, *ε*2/*ε*4, *ε*3/*ε*3, *ε*3/*ε*4, or *ε*4/*ε*4. See Table 2 for genotype frequencies.

The genetic risk score was then computed for each participant by summing the presence or the absence of risk genotypes. That is, each genotype was assigned either “0” for minimally associated genetic risk with cognitive deficits or “1” for a putative association with cognitive deficits. This scoring criterion was based on the literature for each of the three polymorphisms. For* APOE*, the *ε*4 allele is widely considered to be a risk allele for cognitive deficits and decline; so its possession warranted “1” towards the risk score and its absence “0”. For* BDNF*, the* Met* allele is associated with decreased cognitive function; so possession of a* Met* allele resulted in “1” and its absence “0”. For* COMT*, the* Val* allele is considered to be most strongly associated with cognitive deficits, conferring “1” for its possession and “0” in its absence.

In effect, the risk score combines* BDNF Met* carriers into one group to be compared with* Val* homozygotes due to our interest in possession of the* Met* allele at either locus. In a similar fashion, homozygous and heterozygous* COMT Val* carriers were grouped and compared with* Met* homozygotes. Following convention established by the* APOE* literature, *ε*4 allele carriers were grouped and compared with all non-*ε*4 carriers. See Table 2 for risk genotype frequencies for each gene.

The sum of these three component risk genotypes for all three polymorphisms yielded a risk score with a scale of genetic risk values between 0 and 3. An overall risk score value of “0” translates into the absence of any risk genotypes and represents the lowest genetic risk category for decline in cognitive status; “1” represents possession of only one risk genotype, “2” represents possession of two risk genotypes, and “3” represents possession of all 3 risk genotypes and is proposed to represent the highest genetic risk category for decline in cognitive status.

### 2.4. Statistics

First, in order to determine whether any of the three genotypes were individually predictive of cognitive status, hierarchal logistic regression analyses were performed (SPSS Version 22). The demographic variables, consisting of age, gender, and years of education, comprised the 1st block while* BDNF*,* COMT*, and* APOE* genotype were each entered as the 2nd block in separate regression models. In these analyses, the* BDNF* genotype was coded as “1” for* Val/Val*, “2” for* Val/Met*, or “3” for* Met/Met*. In a similar fashion, the* COMT* genotype was coded as “1” for* Val/Val*, “2” for* Val/Met*, or “3” for* Met/Met*. Based on the literature, the independent effect of* APOE* genotype on cognitive status was determined by assigning “1” for non-*ε*4 carriers and “2” for the possession of at least one *ε*4 allele; for subsequent risk score analyses, the* APOE* genotype variable reflects all possible genotypes, coded as “23” for *ε*2/*ε*3, “24” for *ε*2/*ε*4, “33” for *ε*3/*ε*3, “34” for *ε*3/*ε*4, and “44” for *ε*4/*ε*4. These genotype variables were subsequently used as the individual genotype covariates in the genetic risk score analyses.

Hierarchal logistic regression was then performed to determine whether the composite genetic risk score variable (entered as the 3rd block) predicted cognitive status (nonimpaired versus impaired) above and beyond the individual genotypes (all three comprising the 2nd block) and the demographic variables (the 1st block). We report odds ratios (OR) and confidence intervals (C.I.) resulting from these analyses and report *P* values below 0.05.

## 3. Results

### 3.1. Demographics and Covariates

Nine participants were excluded (7 were missing genotype information for all three genes while an additional 2 were missing* APOE* genotype, resulting in an overall failure rate of 7.37% and error rate of 0%). Our final sample size of *n* = 95 was 64.2% female with a mean age of 75.1 years. Allelic frequencies did not significantly differ from Hardy-Weinberg equilibrium for any of the three genes.  Table 3 shows demographic information and MMSE scores for the overall sample as well as between the nonimpaired and impaired participants. Cognitive status (the presence or absence of impairment) was significantly associated with age (OR = 1.113, *P* = .025, and 95% C.I. = 1.014, 1.222) and years of education (OR = .861, *P* = .038, and 95% C.I. = .748, .992) such that higher age and fewer years of education were associated with a higher risk of impairment. Gender was not significantly associated with an increased risk of impairment (OR = .537, *P* = .177, and 95% C.I. = .217, 1.324), but there were a higher proportion of men in the cognitively impaired group and there is evidence of sexual dimorphism of both* BDNF* and* APOE* on risk for AD [46, 47]. Therefore, we included age, gender, and years of education as covariates in all analyses to isolate the effects of* BDNF*,* COMT*, and* APOE* genotype or the genetic risk score on cognitive status. See Table 3.

### 3.2. Association between Individual SNPs and Risk for Cognitive Impairment

Consistent with the literature,* APOEε*4 carrier status was significantly predictive of an increased risk of cognitive impairment (OR = 3.561, *P* = .032, and 95% C.I. = 1.116, 11.365) after controlling variation from age, gender, and education. Neither* BDNF* nor* COMT* genotype was related to cognitive status (*BDNF*, OR = 1.149, *P* = .755, and 95% C.I. = .479, 2.759;* COMT*, OR = 1.074, *P* = .808, and 95% C.I. = .606, 1.903).

### 3.3. Association between Genetic Risk Score and Risk for Cognitive Impairment

**Figure 1:**
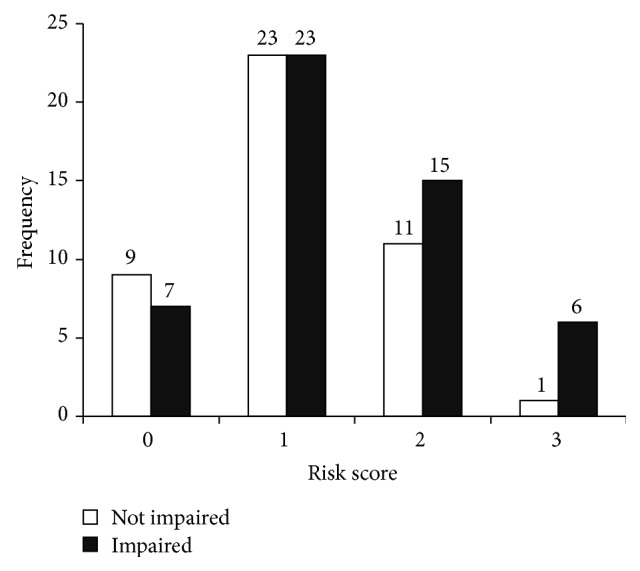
Risk score frequencies between cognitive status categories.

Table 4 shows the frequencies for each genetic risk score value. Each additional point towards the genetic risk score was significantly associated with a nearly 4-fold increased risk of cognitive impairment (OR = 3.824, *P* = .013, and 95% C.I. = 1.333, 10.973) even after including all of the individual gene polymorphisms in the model (see Table 5). Consistent with our hypothesis, a higher genetic risk score representing an increasing number of risk genotypes was predictive of an increased risk of cognitive impairment above and beyond the variation attributable to individual polymorphisms.  Figure 1 shows the distribution of risk scores between cognitive status categories.

Follow-up analyses were performed to determine whether the 4 individuals with dementia were driving the association between the genetic risk score and cognitive impairment. After removing the 4 individuals with dementia from the analysis, the risk score remained significant (OR = 3.138, *P* = .039, and 95% C.I. = 1.059, 9.302) while the individual genotypes remained nonsignificant in the full model, with the exception of* COMT* which reached nominal significance (*BDNF*, OR = .446, *P* = .248, and 95% C.I. = .113, 1.755;* COMT*, OR = 1.977, *P* = .095, and 95% C.I. = .889, 4.396;* APOE*, OR = .899, *P* = .105, and 95% C.I. = .790, 1.022).

## 4. Discussion

We created a genetic risk score to represent the accumulation of risk genotypes of polymorphisms in* BDNF*,* COMT*, and* APOE* to test whether the risk score predicted the presence of late-life cognitive impairment above and beyond that of each individual gene polymorphism. Consistent with our predictions, a higher genetic risk score significantly predicted a higher risk of having cognitive impairment (OR = 3.824, *P* = .013, and 95% C.I. = 1.333, 10.973) when controlling individual polymorphisms in* BDNF*,* COMT*, and* APOE* (see Tables 4 and 5). Congruent with the literature,* BDNF* and* COMT* genotype were not independently predictive of cognitive status, whereas* APOE* *ε*4 carrier status was predictive of cognitive status (OR = 3.561, *P* = .032, and 95% C.I. = 1.116, 11.365). These results demonstrate that the aggregation of multiple risk genotypes into one total risk score predicted the presence or absence of impairment to a higher degree than any of the individual polymorphisms.

The genes under consideration for this study were chosen due to their involvement in general neural function (*APOE*) as well as in processes known to present the earliest deficits in AD such as learning, memory, and executive function (*BDNF* and* COMT*). Polymorphisms in these genes (*BDNF*, rs6265;* COMT*, rs4680; and* APOE*, rs429358/rs7412) have been associated with differential protein function, with possession of each SNP's risk allele (*Met*,* Val*, and *ε*4, resp.) associated with poorer cognitive performance. Our findings support the use of a single genetic risk score to represent the accumulation of genetic influences affecting multiple domains of cognition and brain health in relation to AD, which may be more important in determining cognitive decline than testing individual genotypes. When considered individually, these same genetic polymorphisms contribute small or negligible effects and remain largely undetected by previous GWAs (except* APOE*) but may still represent influential aspects of AD pathology. These results beg the question of the importance of other risk genotypes not assessed here that could also contribute to risk of AD. Additionally, our results complement previous reports of significant interaction between* COMT Val158Met* and* APOEε*4 on risk for AD [48], additive effects of* BDNF Val66Met* and* APOEε*4 on hippocampal activity in healthy older adults [49], and interaction between* BDNF Val66Met* and* APOEε*4 on episodic memory in cognitively healthy older adults [50].

Our study represented a unique population of older adults who underwent a comprehensive cognitive assessment and diagnosis. However, one limitation is the relatively small sample size of our study (*n* = 95). In genetic association studies, hundreds of subjects are genotyped in order to achieve sufficient power to reliably test specific genetic interactions or confirm epistatic effects between genes. However, we still detected an association between higher risk scores and increased odds of being cognitively impaired (Figure 1). Future studies will need to expand these results to larger samples with a higher proportion of cognitively impaired individuals. This will help verify the associations to more clearly examine potential interactions between these gene polymorphisms as well as assess effect sizes and dose-dependent effects of possessing one, both, or no risk allele(s) for each gene considered. The genetic risk score reported here was unable to account for and examine the effects of possessing either one or both risk alleles for each gene without further reducing the number of participants in each risk score category and diminishing the statistical power and interpretation of the results. Finally, an additional limitation of the current study is that we included only individuals of European descent to avoid confounding by population admixture. Future studies should recruit from various racial and ethnic backgrounds to determine whether the genetic and phenotypic trends observed here are present in other races and ethnicities while adequately accounting for population stratification.

In summary, we found that a cumulative genetic risk score across three genes related to cognition, brain function, and risk for dementia (*BDNF*,* COMT*, and* APOE*) significantly predicted late-life cognitive impairment. The risk score was significant above and beyond the effects of each individual genotype. Our findings demonstrate the effectiveness of genetic risk scores and their potential utility as predictors of more genetic variation in cognitive outcomes such as AD than polymorphisms considered individually.

## Figures and Tables

**Table 1:** Genotypes of *BDNF*, *COMT*, and *APOE* SNPs.

SNP	Primer sequence	Primer concentration	Amplicon length	Melt range (°C)
*BDNF* rs6265	FW 5′-GCTTGACATCATTGGCTGAC-3′	375 nM	129	70.0–92.0
	RV 5′-TACTGAGCATCACCCTGGAC-3′			

*COMT* rs4680	FW 5′-TCATCACCATCGAGATCAACC-3′	300 nM	112	70.0–91.0
	RV 5′-TTTTTCCAGGTCTGACAACG-3′			

*APOE* rs429358/rs7412	FW 5′-GGCACGGCTGTCCAAGGA-3′	200 nM	228	80.0–98.0
	RV 5′-GCCCCGGCCTGGTACAC-3′			

**Table 2:** Frequencies of *BDNF*, *COMT*, and *APOE* genotypes and risk allele carriers.

Gene	Genotype	*n*	Risk allele carrier status	*n*
*BDNF *	Val/Val	61	*Met* carriers (risk score value = 1)	34
	Val/Met	33	*Val* homozygotes (risk score value = 0)	61
	Met/Met	1		

*COMT *	Val/Val	23	*Val* carriers (risk score value = 1)	63
	Val/Met	40	*Met* homozygotes (risk score value = 0)	32
	Met/Met	32		

*APOE *	*ε*2/*ε*3	12	*ε*4 carriers (risk score value = 1)	22
	*ε*2/*ε*4	1	Non-*ε*4 carriers (risk score value = 0)	73
	*ε*3/*ε*3	61		
	*ε*3/*ε*4	18		
	*ε*4/*ε*4	3		

**Table 3:** Participant demographics.

Characteristic	All participants (*n* = 95)	Nonimpaired (*n* = 44)	Impaired (*n* = 51)
Age (mean years [SD])	75.1 (5)	73.9 (4.2)	76.1 (5.4)
Completed education (mean years [SD])	14.4 (3.2)	15.1 (2.9)	13.8 (3.3)
Gender (% female)	64.2	70.5	58.8
MMSE (mean [SD])	28.1 (2.1)	28.9 (1.2)	27.5 (2.4)

**Table 4:** Genetic risk score frequencies.

Risk score value	*n*
0	16
1	46
2	26
3	7
Total	95

**Table 5:** Results of genetic risk score controlling individual genotypes.

Variable	OR	*p* value	95% C.I.
*BDNF *	0.369	0.145	0.096–1.410
*COMT *	2.147	0.061	0.966–4.773
*APOE *	0.9	0.093	0.796–1.018
Risk score	3.824	0.013	1.333–10.973
